# The biomedical potential of genetically modified flax seeds overexpressing the glucosyltransferase gene

**DOI:** 10.1186/1472-6882-12-251

**Published:** 2012-12-10

**Authors:** Magdalena Czemplik, Anna Kulma, Karolina Bazela, Jan Szopa

**Affiliations:** 1 Len Pharma, ul. Zamkowa 17, 82-200, Malbork, Poland; 2 Linum Foundation, ul. Stabłowicka 147/149, 54-066, Wrocław, Poland; 3 Faculty of Biotechnology, Wroclaw University, ul. Przybyszewskiego 63/77, 51-148, Wrocław, Poland; 4 Dr Irena Eris Scientific Research Centre, ul. Puławska 107 A, 02-595, Warszawa, Poland

**Keywords:** Flax, Seedcake, Phenylpropanoids, Fibroblasts

## Abstract

**Background:**

Flax (*Linum usitatissimum*) is a potential source of many bioactive components that can be found in its oil and fibers, but also in the seedcake, which is rich in antioxidants. To increase the levels of medically beneficial compounds, a genetically modified flax type (named GT) with an elevated level of phenylopropanoids and their glycoside derivatives was generated. In this study, we investigated the influence of GT seedcake extract preparations on human fibroblast proliferation and migration, and looked at the effect on a human skin model. Moreover, we verified its activity against bacteria of clinical relevance.

**Methods:**

The GT flax used in this study is characterized by overexpression of the glucosyltransferase gene derived from *Solanum sogarandinum*. Five GT seedcake preparations were generated. Their composition was assessed using ultra pressure liquid chromatography and confirmed using the UPLC-QTOF method. For the *in vitro* evaluation, the influence of the GT seedcake preparations on normal human dermal fibroblast proliferation was assessed using the MTT test and the wound scratch assay. A human skin model was used to evaluate the potential for skin irritation. To assess the antimicrobial properties of GT preparations, the percentage of inhibition of bacterial growth was calculated.

**Results:**

The GT seedcake extract had elevated levels of phenylopropanoid compounds in comparison to the control, non-transformed plants. Significant increases in the content of ferulic acid, *p*-coumaric acid and caffeic acid, and their glucoside derivatives, kaempferol, quercitin and secoisolariciresinol diglucoside (SDG) were observed in the seeds of the modified plants. The GT seedcake preparations were shown to promote the proliferation of normal human dermal fibroblasts and the migration of fibroblasts in the wound scratch assay. The superior effect of GT seedcake extract on fibroblast migration was observed after a 24-hour treatment. The skin irritation test indicated that GT seedcake preparations have no harmful effect on human skin. Moreover, GT seedcake preparations exhibited inhibitory properties toward two bacterial strains: *Staphylococcus aureus* and *Escherichia coli*.

**Conclusions:**

We suggest that preparations derived from the new GT flax are an effective source of phenylopropanoids and that their glycoside derivatives and might be promising natural products with both healing and bacteriostatic effects. This flax-derived product is a good candidate for application in the repair and regeneration of human skin and might also be an alternative to antibiotic therapy for infected wounds.

## Background

Wound healing is a complex process that involves three main overlapping phases: inflammation, proliferation and tissue remodeling [1]. It has been shown that many growth factors, cytokines and proteases are crucial for tissue repair after damage. The involvement of fibroblasts and keratinocytes is necessary to achieve total wound closure. Both types of cell migrate and undergo differentiation to restore the skin barrier [2]. Adverse effects, such as possible pathogenesis, infections or ineffective healing, may significantly delay the process of wound closure. Therefore, the search for alternative compounds that improve wound healing is of great interest.

Recently, numerous research groups have reported on the traditional use of plants for wound healing. Annan and Houghton showed the significant effects on the growth of human dermal fibroblasts of extracts from *Gossypium arboreum* and *Ficus asperifolia*, and also reported on their protective effects on these cells against oxidative damage [3]. The ethanol root extract of *Ixora coccinea* improved wound contraction and exhibited antibacterial activity [4]. Increased proliferation and migration of keratinocites were observed in an *in vitro* study using aqueous extracts from the leaves of *Chromolaena odorata*[5]. Phenolic compounds, i.e. flavonoids and phenolic acids, are known to regulate the normal human dermal fibroblast genes involved in antioxidant defense, the inflammatory response and cell renewal [6].

Recent research has focused on identifying the potential plant-derived agents that influence wound healing. The biological activity of plant extracts has been known for many years, as plants produce a wide range of phytochemicals. One source of such bioactive compounds is flax (*Linum usitatissimum*), which is widely distributed in Mediterranean and temperate climate zone. Flax seeds are known to play an important role in the food industry and health care. The known beneficial properties of flax are mainly associated with its oil, but a great amount of bioactive phytochemicals remains in the seedcake (the leftovers of the flax seeds after oil extraction). Flax seedcake contains phenolic acids, flavonoids, and other phenylopropanoids that are known to possess a wide range of biological activities and thus have a beneficial influence on human health [7]. The valuable effects of phenylopropanoids are mainly due to their antioxidant properties. They prevent cardiovascular diseases, arteriosclerosis, cancers, inflammations and diabetes [8-10]. The seedcake is also a rich source of secoisolariciresinol diglucoside (SDG), the precursor of lignans, known to inhibit cancer cell proliferation and growth [11]. SDG also possesses anti-bacterial, anti-fungal and anti-viral properties [12].

The beneficial features of flax products could clearly be enhanced if the secondary metabolite accumulation in the plant organs could be increased. In an attempt to increase the level of phenylpropanoid compounds in flax, a genetically modified flax type (named GT) with an elevated level of phenylopropanoids and their glycoside derivatives was generated. GT overexpresses the glucosyltransferase gene derived from *Solanum sogarandinum* in its seeds. Glycosyltransferases are enzymes that transfer activated sugar donors to other metabolites. In plants, phenylopropanoids can be acceptors of sugar moieties and are often converted to their glycoconjugates, which are then accumulated and compartmentalized in vacuoles [13]. Glycosylation of phytochemicals is known to alter their regulatory properties by enhancing water solubility and increasing stability. Therefore, the performed modification aimed to increase the amount and stability of phenylopropanoids in flax seeds. In this study, we estimated the effect of GT flax seedcake extracts on the growth and migration of human dermal fibroblasts, and we determined the potential for skin irritation using a human skin model.

## Methods

### Plant material

Flax seeds (cv. Linola 947) were obtained from the Flax and Hemp Collection of the Institute of Natural Fibers, Poland. Transgenic plant construction and selection was performed as previously described by Lorenc-Kukuła *et al*. [14]. The GT plants (previously called UGT plants) overexpress SsGT1 (EMBL/GenBank accession no. AY033489) from *Solanum sogarandinum* under a seed- specific napin promoter (EMBL/GenBank, accession no. J02798) and the OSC terminator. In this study, the third generation of GT transgenic plants line #4 (GT4) was grown in a field, and the seeds were harvested 3 months after sowing, in parallel, the control plants (cv. Linola 947, obtained from the Flax and Hemp Collection of the Institute of Natural Fibers, Poland) were grown in the same location (Lower Silesia, Poland) in the same year. GT4 and Linola seeds were pressed to obtain oil on an industrial warm gear oil press (Oil PressDD85G – IBG Monoforts Oekotec GmbH& Co). The GT4 and Linola seedcakes were collected and used for further experiments.

### Identification and quantification of phenylpropanoids in flax seedcake extracts

A 0.25 g sample of GT4 flax seedcakes was extracted three times with 1.5 mL of 80% methanol (v/v) for 10 min at 80°C. Prior to extraction, the seedcakes were defatted with hot hexane. The extract was centrifuged and evaporated at 40°C under a vacuum and then resuspended in water and subjected to alkaline hydrolysis (1 mL, 0.3 M aqueous sodium hydroxide) for 2 days at room temperature followed by neutralization using 2 M hydrochloric acid [15]. The extract was analyzed on a Waters Acquity UPLC system with a 2996 PDA detector, using an Acquity UPLC column BEH C18, 2.1100 mm, 1.7 μm. The mobile phase was A = acetonitrile and B = 20 mM ammonium formate, pH 3, in a gradient flow: 1 min, 10%/90% A/B, 2–6 min gradient to 40%/60% A/B, and 7 min gradient from 40% to 100% A with a 0.4 mL/min flow rate. The compounds were measured at 280 and 320 nm.

The identity of the components was confirmed by LC-MS analysis on a Waters Aquity UPLC-QTOF system using BEH C18, 2.1 150 mm, 1.7 μm The mobile phase was A = acetonitrile and B = 0.1% formic acid, in a gradient flow: 1 min, 10%/90% A/B, 2–10 min gradient to 80%/20% A/B, and 12 min gradient from 80% to 100% A with a 0.4 mL/min flow rate. The MS spectra were recorded in ESI positive mode for 13 min in 50–800 Da range. The parameters were: nitrogen flow 800 L/h, source temperature 70°C, desolvation temperature cone 400°C, capillary voltage 3.50 V, sampling cone 30 V, cone voltage ramp 40–60 V, scan time 0.2 sec. The obtained spectra were compared to those of known standards and data in the literature.

### The components of GT4 seedcake preparations

GT4 seedcake extract was adequately diluted for the purpose of fibroblast treatment. The final concentration of phenylopropanoids added to the cell culture is listed in Table 1. Prior to fibroblast treatment, the GT4 preparations were sterilized by filtration through an Acrodisc 0.22 μm filter (Gelman Sciences, Ann Arbor, MI, USA).

**Table 1:** Biochemical composition of GT4 seedcakes preparations used for cell in vitro studies

**Preparation no**/**compound**	**SDG**	**Ferulic acid**	***p***-**coumaric acid**	**Ferulic acid glucoside**	***p***-**coumaric acid glucoside**	**Caffeic acid**	**Caffeic acid glucoside**
	**(mg/mL)**	**(mg/mL)**	**(mg/mL)**	**(mg/mL)**	**(mg/mL)**	**(mg/mL)**	**(mg/mL)**
**1**	0,5	0.016	0.011	0.22	0.086	0.00022	1.22
**2**	1	0.032	0.022	0.44	0.172	0.00045	2.45
**3**	2	0.064	0.044	0.88	0.344	0.0009	4.91
**4**	4	0.128	0.088	1.76	0.688	0.0018	9.83
**5**	6	0.192	0.132	2.64	1.032	0.0027	14.74

1- 5 are numbered preparations used in this study.

### Antioxidant activity of GT4 preparations

Radical scavenging activity of the GT4 seedcake preparations and pure compounds was determined using the stable free radical 2,2’-diphenylpicrylhydrazyl (DPPH) method. A quantity of 40 μl of each test solution (GT4 preparation No. 2, 3 and 4, 1 mM ferulic acid, 1 mM p-coumaric acid, 1 mM quercetin, 1 mM rutin, 1 mM vitamin C) was mixed with 1000 μl of a freshly prepared DPPH-methanol solution (0,1 mM) and allowed to stand for 30 min at room temperature. The optical densities of the resulting solutions were read at 517 nm using a Cary 50 Conc UV/vis spectrometer.

### Cell culture

Normal human dermal firbroblasts (NHDF) were obtained from Laboratory of Cell Pathology, Faculty of Biotechnology at Wrocław University, Poland. The NHDF cells were maintained at 37°C, 5% CO_2_ in Minimum Essential Medium Alpha (α-MEM, Institute of Immunology and Experimental Therapy, Polish Academy of Science, Poland) supplemented with 10% fetal bovine serum (FBS, Lonza, USA), 1% L-glutamine (Invitorgen, USA) and 1% antibiotic mixture (Invitorgen, USA). NHDF used in this study were between the third and seventh passages. 24 h prior to the treatment, the medium was replaced with α-MEM containing 0.5% FBS. The 0.5% FBS concentration is a maintenance dose needed for the production of healthy cells. It does not significantly stimulate proliferation of cells.

### Fibroblast microscopy

Cells were seeded in a 24-well plate at a concentration of 1 × 10^4^ cells/mL, and after 24 h GT4 seedcake preparations (#1-#5) were added to the plate. Non-treated cells served as a control. To assess the effect of the GT4 seedcake extracts on the growth of the cells, microscopic observations were performed after 24 h and 48 h using a transmitted light phase contrast microscope equipped with × 10 and × 100 objectives (Axiovert 40 CFL, ZEISS).

### Cell proliferation assay

Cells were seeded in a 24-well plate at concentration of 1 × 10^4^ cells/mL, and after 24 h GT4 seedcake preparations (#1-#5) were added to the plate. Non-treated cells served as a control. To assess the proliferation potential, NHDF cells were incubated and assayed after 72 h. After this period of treatment, 50 μL of MTT stock solution (4 mg/mL) was added to each well to give a total reaction volume of 550 μL. After incubating for 4 h, the medium with MTT solution was removed from the plate. The formazan crystals in each well were dissolved in 500 μL of DMSO and incubated for 30 min with gentle shaking. The absorbance at 540 nm was read on an Asys UVM340 Microplate Reader (Biochrom, UK). The MTT assay was performed in triplicate. The results were presented in % as a reference to the control (100%).

### *In vitro* wound scratch assay

Wound-healing properties were evaluated using the *in vitro* scratch assay [16], which measures the expansion of a cell population on surfaces. NHDF cells were seeded in a 24-well plate at concentration of 1 × 10^4^ cells/mL, and maintained to nearly confluent cell monolayers. Next, a linear wound was generated in the monolayer with a sterile 100-μL plastic pipette tip. Any cellular debris was removed by washing with phosphate buffer saline (PBS). α-MEM containing 0.5% FBS, 1% L-glutamine (Invitrogen, USA) and 1% antibiotic mixture (Invitrogen, USA) was added to the plate. Then, the GT4 seedcake preparations (#1-#4) were added. Non-treated cells served as a control. To estimate the relative migration of NHDF cells, three representative images of the scratched areas from each well were photographed using a transmitted light phase contrast microscope equipped with × 10 and × 100 objectives (Axiovert 40 CFL, ZEISS). Wound closure was determined as the difference in expansion area at 0, 24 and 48 h. The experiments were performed at least in triplicate. The data were analyzed using TScratch software.

### Skin irritation test

An *in vitro* skin irritation test was performed according to the MTT Effective Time-50 (ET-50) protocol developed at MatTek Corporation with the use of the EpiDerm skin irritation test. This test allows the assessment of skin irritation due to cosmetic ingredients and ready-made products. 100 μL of three preparations of GT4 seedcake extracts (#2, #4 and the concentrated, non-diluted preparation #36) were put on the surface of the epidermis model. After an incubation period of 2, 5 or 18 h, cell viability was assessed using the MTT colorimetric test (described above). Skin irritation potential is predicted if the remaining relative cell viability is below 50%. The experiment was performed in duplicate.

### Antimicrobial activity

*Staphylococcus aureus* strain ATCC 6538 and *Escherichia coli* strain ATCC 10536 were used as indicators for antimicrobial testing and were obtained from the in-house culture collection of the Microbiology Department at the University of Wrocław, Poland. Bacteria were grown at 37°C in Luria-Bertani (LB) medium under shaking conditions. Diluted (100-fold in LB) overnight cultures of all of the tested bacteria strains (150 μL) were incubated in 96-well plates at 37°C, shaking at 600 rpm with a previously prepared seedcake extract of GT4 flax. The volumes contained the standardized proportions of 0.5 to 25 mg/ml SDG. OD_600_ was monitored at 4, 6 and 12 h in an Asys UVM340 Microplate Reader (Biochrom, UK).

## Results and discussion

### Identification and quantification of phenylopropanoids in seedcake extracts of GT flax

The overexpression of the glycosyltransferase gene under the seed-specific napin promoter aimed to increase the levels of phenylopropanoid compounds in flax seeds. GT plants (formerly named UGT plants) were previously described and characterized by Lorenc-Kukuła *et al*. [14]. Transgenic line #4 was analyzed (GT4) as it has the highest expression of introduced gene. In order to identify and analyze the content of the GT4 flax seedcake, methanol extracts were prepared. The quantitative analysis of the extracts of GT4 and the control plants (Linola) was performed with UPLC and the identity of the components was confirmed by UPLC-QTOF analysis. In the seedcakes of GT flax, we indentified lignan (SDG) and phenolic acids and their glucoside derivatives (Table 2).

**Table 2:** UPLC-QTOF identification of phenylpropanoids of seedcake extracts

**Compound**	**RT (min)**	**PDA max (nm)**	**MS Ions**
Caffeic acid glucoside	1.70	240, 290 (A max), 317 (sh)	**163**[M-Glu-H_2_O + H]^+^
			181[M-Glu + H] ^+^,
			343 [M-H_2_0 + H] ^+^
*p*-coumaric acid glucoside	1.81	231, 296 (A max)	**147**[M-Glu-H_2_O + H]^+^
			165[M-Glu + H] ^+^,
			327 [M-H_2_0 + H] ^+^
Ferulic acid glucoside	2.27	237, 291 (A max), 316 (sh)	**177**[M-Glu-H_2_O + H]^+^
			195[M-Glu + H] ^+^,
			357 [M-H_2_0 + H] ^+^
Caffeic acid	3.39	240, 297 (sh), 322 (A max)	**163**[M-H2O + H] ^+^,
			181[M + H] ^+^
*p*-coumaric acid	5.25	230, 300 (sh), 309(A max)	**147**[M-H2O + H]^+^,
			165[M + H] ^+^
Secoisolariciresinol diglucoside (SDG)	6.26	233, 281 (A max)	**327**[M-2Glu + H]^+^,
			525[M-Glu + H_2_0 + H]^+^,
			687[M + H]^+^
Ferulic acid	6.43	246, 297 (sh), 323 (A max)	**177**[M-H2O + H] ^+^,
			195[M + H] ^+^

The UPLC analysis revealed a significantly higher content of secoisolariciresinol diglucoside (SDG) in the seedcakes of the modified flax GT4 in comparison to the control (36.8 mg/gFW). SDG is the main lignan of flax seeds and is known to possess many favorable effects on human health along with anti-bacterial, anti-viral and anti-fungal properties. The GT4 seedcake extracts were characterized by an increased level of phenolic acids, which are known to exhibit significant antioxidative properties. The main representatives of the phenolic acid family were *p*-coumaric, ferulic and caffeic acids and their glucoside derivatives. GT4 seedcakes showed an increase in the contents of phenolic acids and especially in those of their glucoside derivatives. The identification of the flax seedcake components by UPLC-QTOF analysis confirmed the presence of the caffeic acid glucoside, which has thus far not been widely reported. The respective accumulation of glucosides of *p*-coumaric, ferulic and caffeic acid was 19702 μg/FW, 1175 μg/FW and 90636 μg/FW. This strategy of modification, i.e. overexpression of glucosyltransferase in seeds, appeared to be effective for increasing the pool of stable phenolic glucosides and thus broadening the application possibilities for flax (Table 3). The elevated level of phenylpropanoids in GT4 flax occurs yearly in comparison to the control, so it is suggested that the above modification is stable throughout generations in flax. Seedcakes, which are rich in phenylpropanoids, are known to possess significant antioxidative properties [17,18]. It is suggested that the presence of phenolic acids in particular significantly increases the antioxidative potential of flax seedcakes, as the relationship between these metabolites and antioxidant activity is known [19,20]. The extract of GT4 seedcakes exhibited significant radical scavenging activity against the stable DPPH free radical. GT4 seedcake preparations # 1, #2, #3 and #4 showed better antioxidant activity than 1 mM ferulic acid, 1 mM *p*-coumaric acod and 1 mM vitamine C. The activity of GT4 seedcake preparations was comparable to that of 1 mM quercetin and 1 mM rutin (data not shown). Therefore, flax seedcakes are potentially a great raw material for new products for biomedical applications, and we suggest that GT4 seedcake preparations could be used as strong antioxidants ameliorating the wound-healing process. However limitation of the present study comprises determination of biomedical potential only of modified GT4 seeds. As the previous comparison of modified and control flax indicated that the modified flax is characterized with much better qualities, we focus on analyzing only the GT4 flax. Especially, the greatest achievement was the significant improvement of GT4 flax resistance, thus productivity and also the increased levels of different phenylpropanoids. These qualities make GT4 flax the better source of such a metabolites, mainly because its cultivation is economically profitable.

**Table 3:** Composition of the seedcake extracts of transgenic GT4 and control (Linola) flax

**Compound**	**Linola (μg/FW)**	**GT4 (μg/FW)**
Secoisolariciresinol diglucoside (SDG)	19095 ± 118	36880 ± 13
*p*-coumaric acid	51 ± 2.6	87 ± 3.6
*p*-coumaric acid glucoside	12621.57 ± 23	19702.4 ± 613
Ferulic acid	805.1 ± 57	1175 ± 62
Ferulic acid glucoside	87570 ± 34	179386 ± 942
Caffeic acid	28.2 ± 2.0	16.6 ± 5.5
Caffeic acid glucoside	64248 ± 37	90636 ± 76

Moreover the previous preliminary research on the cell cycle of fibroblasts treated with seedcake extracts of Linola seeds and GT4 seeds indicated that the percentage of proliferating fibroblast was higher for GT4 preparation treated cells than for Linola preparation treated cells (data not shown). For these reason GT4 modified fax has been chosen as an object for further studies, presented in this manuscript.

### Effects of GT4 seedcake preparations on the growth and proliferation of normal human dermal fibroblasts

Due to the reported antioxidant properties of flax seed constituents, it was suggested that GT4 seedcakes might be a great source of health-promoting compounds. To investigate their effect on human dermal fibroblasts and a human skin model, five different dilutions of GT4 seedcake extract were prepared (#1-#5). The detailed data for the phenylopropanoid contents in the GT4 seedcake extracts used for our *in vitro* cell culture experiments are presented in Table 1.

**Figure 1:**
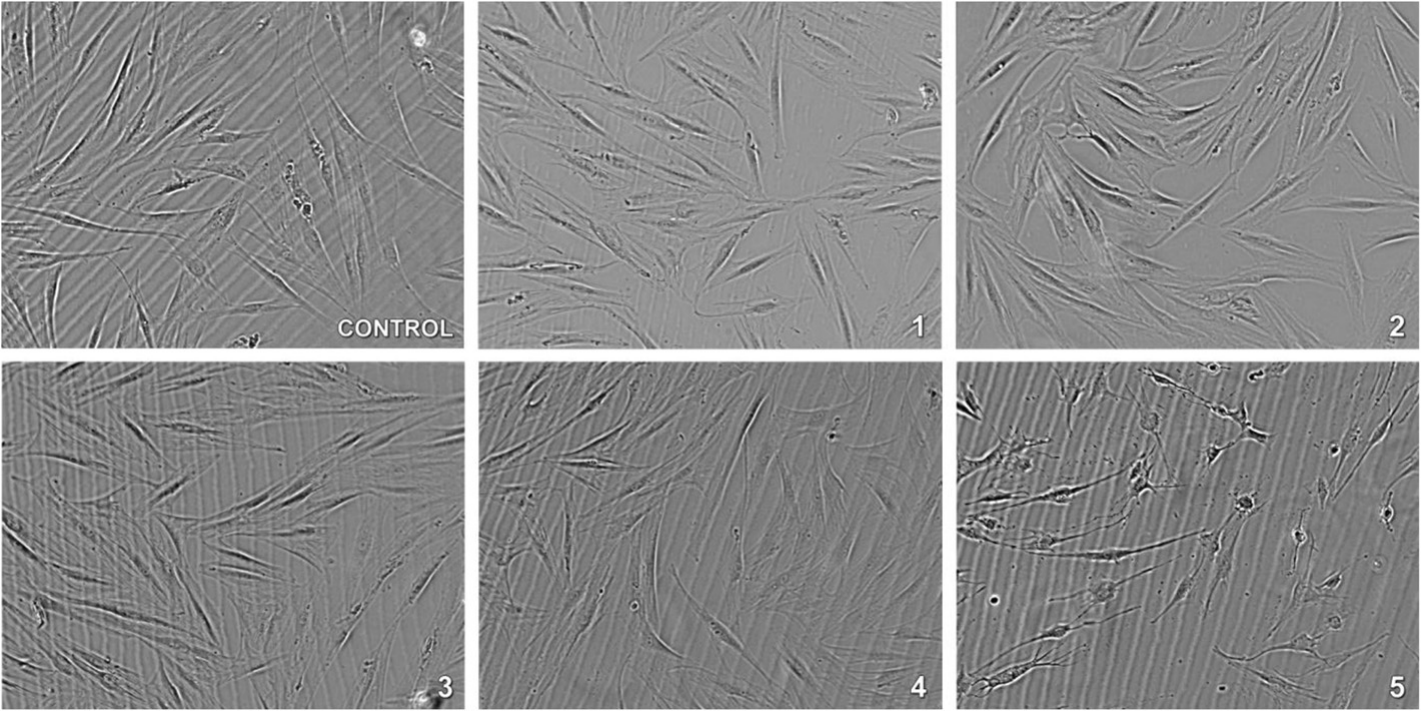
Light microscope images of NHDF cells (×100) treated with GT4 seedcake preparations #1-#5 (1-5) and non-treated control cells (C) after 24 h incubation.

We assessed the growth of normal human dermal fibroblast (NHDF) cells treated with GT4 seedcake preparations #1-#5. The NHDF cells were examined microscopically to evaluate the effects on their phenotype and growth after 24 and 48 h. Similar effects were observed at both times. There was no visible difference between the control cells and the cells treated with GT4 seedcake preparations #1, #2, #3 and #4. The NHDF cells exhibited the normal growth and phenotype. By contrast, significant differences were observed after treatment with GT4 seedcake preparation #5 (Figure 1). The NHDF cells exhibited an altered and impaired phenotype and inhibited growth.

**Figure 2:**
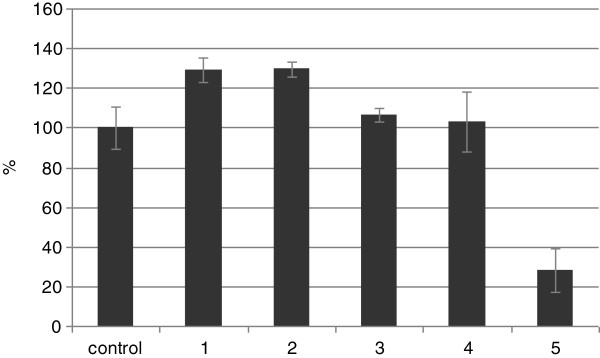
**Proliferation percentage of NHDF cells in *in vitro *MTT test.** The numbers 1-5 correspond to the NHDF cells treated with GT4 seedcake preparations #1-#5. The MTT assay was performed in triplicate after 72 h of incubation.

Furthermore, the proliferation of fibroblasts treated with the five GT4 seedcake preparations were assessed using the MTT test. Of the five tested preparations, #1 and #2 exhibited a positive effect on NHDF proliferation. In both samples, a 30% increase in proliferation was observed. Preparations #3 and #4 had no effect on NHDF proliferation (a slight increase of 7% and 3%, respectively), while preparation #5 significantly decreased (72%) the proliferation potential of the fibroblasts (Figure 2).

During wound healing, the balance between oxidative and antioxidative processes is disturbed, which results in excessive free radical generation. This oxidative stress is a crucial factor in wound progression and pathogenesis [21]. It promotes the damage of many cellular components and mechanisms and causes fibroblast apoptosis [22]. In chronic wounds, the endogenous antioxidants are not sufficient for free radical neutralization, so providing additional antioxidants might restore oxidative homeostasis [23].

It is suggested that plant-derived compounds of the phenylopropanoid pathway have a positive influence on wound healing. There are some reports confirming the beneficial influence of phenolic plant extracts on fibroblasts. Kim *et al*. showed the enhanced proliferation of human skin fibroblasts treated with *Ginkgo biloba* extract. This proliferative effect is due to the action of phenylopropanoids [24]. It was also reported that a leaf extract of *Wedelia trilobata* displayed a stimulatory effect on fibroblasts and promoted the synthesis of collagen [25]. Therefore, it was suggested that the extract from seedcakes of modified flax with elevated levels of phenylpropanoids might improve the proliferation and migration of human fibroblasts.

Indeed, our study revealed that GT4 seedcake preparations enhanced normal human dermal fibroblast proliferation by 30%. Such a rate of increase is not spectacular, but a significant increase in the proliferation potential of fibroblasts is not favorable during wound healing, as it promotes overgrowth of connective tissue and scar formation.

On the other hand the relationship between fibroblasts growth in *in vitro* cultures and *in vivo* wound closure are not elucidated yet, so the *in vivo* effect to of GT4 seedcake preparation extract might be different and eventually less significant. Moreover, the growth and mobility of fibroblasts depends on the presence of the extracellular matrix, so these effects are still to be elucidated in *in vivo* experiments. Of all the preparations tested, the one with the highest phenylpropanoid content (#5) exhibited negative effects on NHDF cells, which was observed in the microscopic study and proliferation test. It is estimated that such a high concentration of phenylpropanoids might result in the predominance of pro-oxidative processes over antioxidative processes, probably due to the reduction of chelated Fe^3+^ to Fe^2+^ and the subsequent radical generation by metal-induced oxidation [26]. This might partially explain the toxic effect of preparation #5 to cells. Therefore, preparation #5 was excluded from the following experiment.

### Migration of GT seedcake-treated fibroblasts in wound scratch assay

One of the crucial processes for wound closure is the migration of fibroblasts. They migrate to the wound, form granulation tissue, and create the basis for the wound closure [27]. The migration potential of NHDF cells treated with GT4 seedcake extracts was assessed in the scratch assay, a method that mimics the migration of cells *in vivo*[28] and is commonly used to study cell migration *in vitro*. For the experiment, we chose GT4 seedcake preparations #1, #2, #3 and #4, which exhibited no negative effect on fibroblast growth or proliferation. The observations of fibroblast migration were performed using light microscopy and the migration was determined as the expansion of a cell population on surfaces after 24 and 48 h.

**Figure 3:**
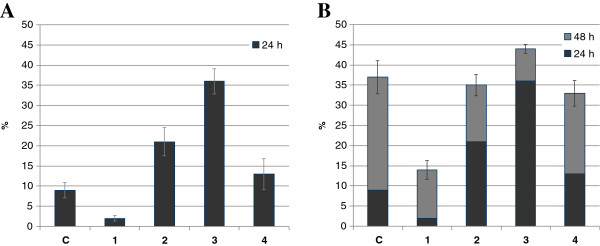
**Migration of fibroblasts treated with GT seedcake preparations #1-#4 in the wound scratch assay.** Panel **A** represents the results after 24 h treatment, and panel **B** after 24 and 48 h treatment.

The wound scratch assay showed differences between the NHDF cells treated with GT4 seedcake preparations and the control cells after 24 h of incubation. The highest migration potential was observed for NHDF cells treated with preparations #2 and #3 (21 and 36%, respectively) while the control cells exhibited 9% surface expansion (Figure 3A). No further significant differences after 48 h treatment could be shown other than a slight increase in migration with the treatment with preparation #3 (Figure 3B). Preparation #4 had no effect on the migration of NHDF cells and preparation #1 slowed the migration of the fibroblasts. The microscope observations are shown in the supplementary data (Additional file 1: Appendix A).

The presented results suggest the GT4 seedcake preparations are effective in enhancing migration of NHDF cells, but only during the first 24 h. The phenylpropanoids in GT4 seedcake are thus thought to be responsible for the increase of NHDF cell migration, but their action is time-limited. It is also of note that the highly concentrated GT4 seedcake extract #4 was practically ineffective in the acceleration of wound closure. Preparation #1 slowed migration of NHDF cells suggesting that the lowest concentration of phenylpropanoids night not enhance the mobility of skin fibroblasts but enhance their growth. Migration is a complex process that involves coordinated polymerization, adhesion, actin polymerization, and movement to the membrane in the migration direction. To find out which phases are affected by GT4 seedcake preparations, further experiments must be performed. Fibroblast proliferation and migration are independent mechanisms, which might partially explain the different effects of GT4 seedcake preparations on these two processes. Similarly, the mechanism of influence on NHDF proliferation remains to be elucidated.

### Evaluation of skin irritation caused by GT seedcake preparations

To better assess the application potential of GT4 seedcake preparations, the skin irritation test was performed with use of EpiDerm, a three-dimensional human skin model. This is a fully developed epidermis with a functional stratum corneum, i.e. with keratinocytes in various stages of differentiation. It is commonly used to assess the irritation potential of dermally applied substances. This test can be applied as an alternative for evaluating dermal irritation prior to clinical tests. The GT4 seedcake preparations were directly applied to the stratum corneum of this air-lifted, highly differentiated culture. The *in vitro* skin irritation evaluation was based on the MTT test and assessed 2, 5 and 18 h after application of the preparations.

**Figure 4:**
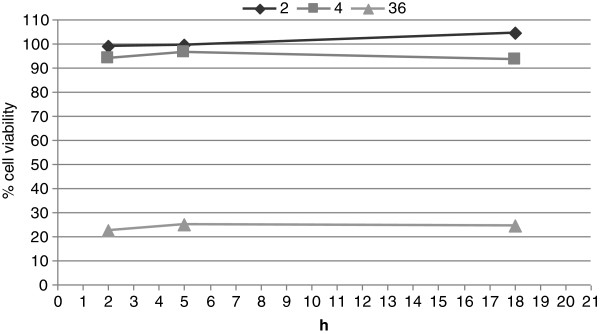
**Skin irritation potential of GT4 seedcake preparations on reconstructed human epidermal model EpiDerm.** The irritation potential was determined for preparations #2, #4 and non-diluted #36 after 2, 5 and 18 h of treatment, The experiment was performed in duplicate.

Preparations #2 and #4 showed no irritating effects on the human skin model (Figure 4). The cell viability after the application of preparation #2 was 99, 100 and 105% at 2, 5 and 18 h, respectively, and after the application of preparation #4 was 94, 97 and 94% at 2, 5 and 18 h, respectively.

Additionally, we tested the effect of non-diluted GT4 seedcake extract (#36) on the human skin model. The cell viability significantly decreased, which indicates the concentrated GT4 seedcake extract possesses properties that irritate the skin.

We suggest that high concentrations of phenylopropanoids in GT4 seedcake preparations are toxic to keratinocytes. Preparations #1-#4 appeared to be safe for dermatological applications, but caution must be taken if using highly concentrated extracts, since proliferation of fibroblasts can be affected and an irritation reaction can occur.

### Antimicrobial properties of GT4 seedcake preparations

Wounds are exposed to the external environment and are thus prone to attack by microbes, which delay the wound-healing process. Therefore, the antimicrobial activity of GT4 seedcake extracts was assessed on strains of common pathogens of human skin. According to the literature, 27% of chronic wounds are infected with *E*. *coli* and 57.1% with *S*. *aureus*[29]. Thus, preparations dedicated to improve the healing of chronic wounds should also be antibacterial agents.

**Figure 5:**
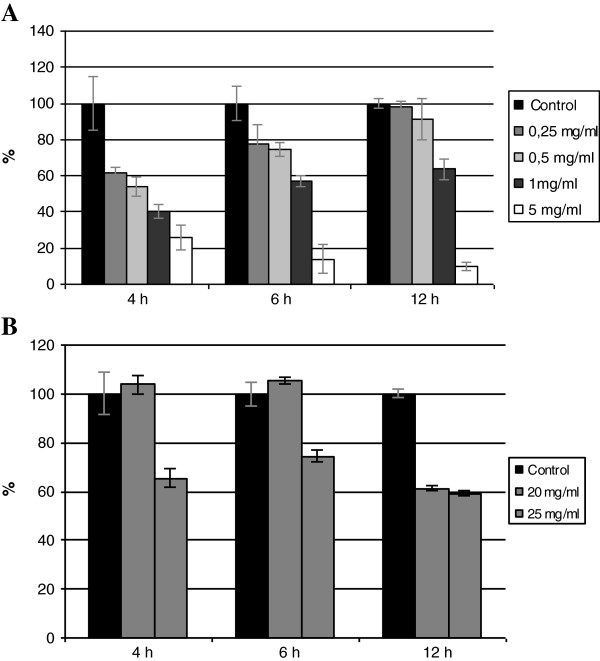
**Antibacterial activity of GT4 seedcake extracts against*S. aureus *(A) and *E. coli *(B). ** The results represent the percentage of bacterial growth in the presence of the seedcake extract (the average of three independent repeats) compared to the control (non-treated culture of *S*. *aureus* or *E*. *coli*). The extract concentration is expressed in mg of SDG per mL.

As the main constituent of seedcake extract is SDG, its content was used as the reference in this experiment. GT4 seedcake extracts were found to inhibit the growth of the two bacterial strains. The bacterial growth inhibition was assessed after 4, 6 and 12 h of GT4 seedcake extract treatment. *S*. *aureus* appeared to be more susceptible than *E*. *coli*. *S*. *aureus* growth was inhibited by a low concentration of GT4 seedcake extract (0.25 mg/mL of SDG) and at 5 mg/mL of SDG content, the *S*. *aureus* growth was significantly inhibited (90% after 12 h of treatment; Figure 5A). A much higher concentration of GT4 seedcake extract was needed to inhibit the *E*. *coli* growth. 20 mg/mL and 25 mg/mL respectively caused 38 and 40% inhibition after 12 h of treatment (Figure 5B). Many studies indicate that most of the active plant extracts show their highest activity against Gram-positive strains such as *S*. *aureus*[30,31]. It is suggested that Gram-positive bacteria are generally more sensitive to plant extracts than Gram-negative bacteria. This was consistent with our observations. Moreover, it is estimated that a mixture of phenylpropanoids is a more effective antibacterial agent than a pure, single compound. The results suggest that GT4 seedcake preparations can be applied in infected wounds or skin infections caused by *S*. *aureus*.

## Conclusions

Our findings demonstrate that genetic engineering can be used to improve the qualities of flax seeds. Overexpression of the glucosyltransferase gene in seeds increased the content of phenylopropanoids and their glucoside derivatives. Flax seedcakes rich in these compounds might be a promising source of products that are beneficial for human health. The positive influence on fibroblast proliferation and migration and the bacteriostatic effects suggest that GT4 seedcake preparations might be a good candidate for the repair and regeneration of human skin and an alternative to antibiotic therapy of infected wounds. Moreover, our results show that these preparations are safe for use on the skin. Such new application possibilities of flax and especially their biomedical relevance can contribute to the renewal of flax cultivation worldwide.

## Abbreviations

GT: Flax type overexpressing glucosyltransferase gene derived from *Solanum sogarandinum* in flax seeds; GT4: Line # of flax type overexpressing glucosyltransferase gene derived from *Solanum sogarandinum* in flax seeds.

## Competing interests

The authors declare that they have no competing interests.

## Authors’ contributions

MC was responsible for the concept, data collection, analysis and interpretation and drafting of the manuscript. AK performed the UPLC-QTOF analysis and made substantial contribution to the concept and design of the manuscript. KB performed the skin irritation potential assay. JS contributed to the interpretation of the data and revised it critically, and gave final approval of the version to be published. All authors read and approved the final manuscript.

## Pre-publication history

The pre-publication history for this paper can be accessed here:

http://www.biomedcentral.com/1472-6882/12/251/prepub

## Supplementary Material

Additional file 1 **Appendix A.** Migration of fibroblasts treated with GT seedcake preparations #1-#4 in wound scratch assay after 24 h and 48 h treatment. For the purpose of improved visualization NHDF cells were marked in red. The observations were performed in light microscope (×100). Click here for file
