# Supplementary material for: The biomedical potential of genetically modified flax seeds overexpressing the glucosyltransferase gene
Source: BMC Complement Altern Med. 2012 Dec 10;12:251. doi: 10.1186/1472-6882-12-251 (PMC3640942; doi:10.1186/1472-6882-12-251)
Supplement: Additional file 1 — Appendix A. Migration of fibroblasts treated with GT seedcake preparations #1-#4 in wound scratch assay after 24 h and 48 h treatment. For the purpose of improved visualization NHDF cells were marked in red. The observations were performed in light microscope (×100). [file 1472-6882-12-251-S1.doc]

8. Appendix A


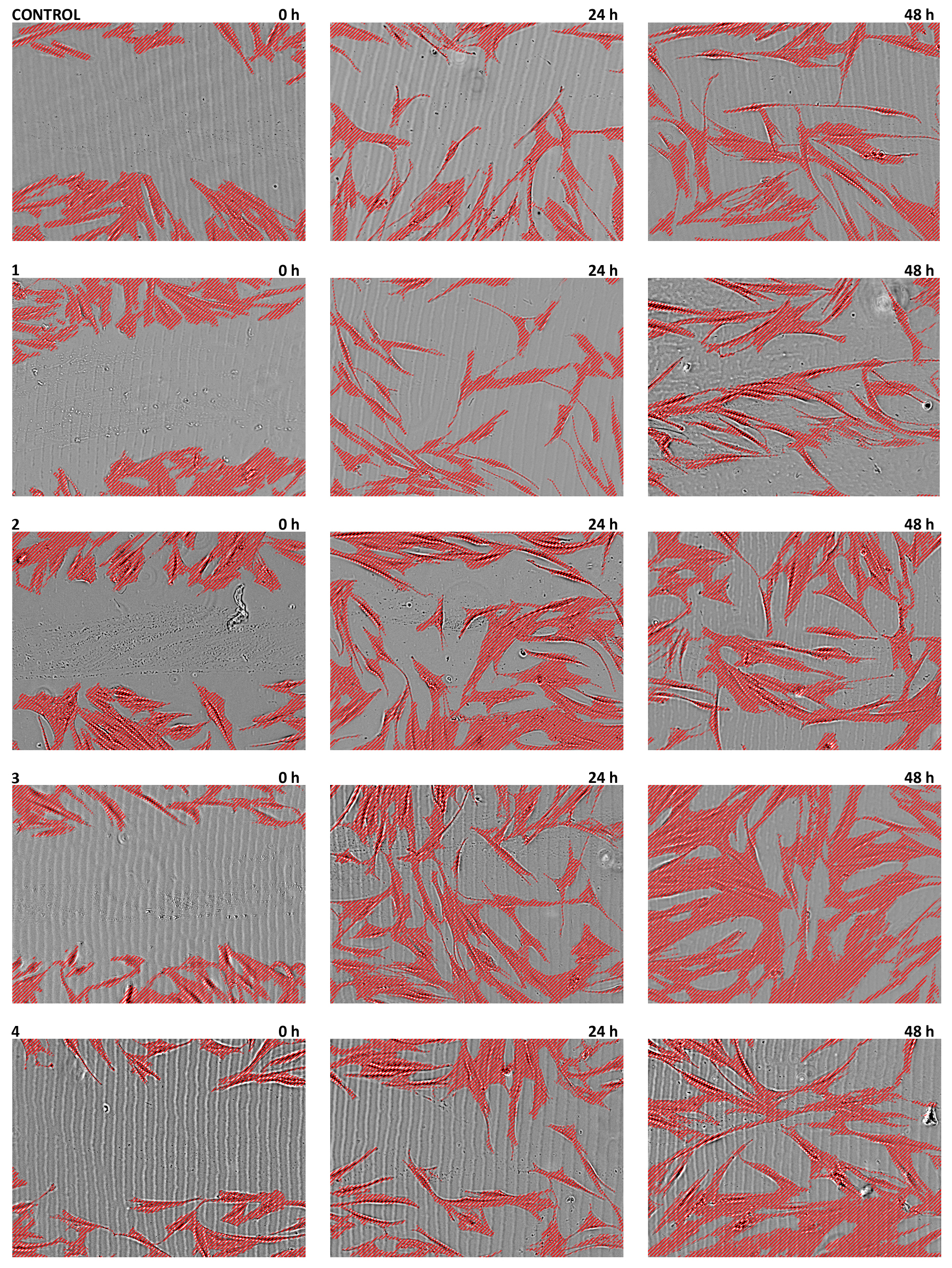


Migration of fibroblasts treated with GT seedcake preparations #1-#4 in wound scratch assay after 24 h and 48 h treatment. For the purpose of improved visualization NHDF cells were marked in red. The observations were performed in light microscope (×100).
